# Kidney Disease as Risk of In-Hospital Mortality in Patients With Acute Coronary Syndrome

**DOI:** 10.7759/cureus.19557

**Published:** 2021-11-14

**Authors:** Gustavo Lenci Marques, Noessa Hiromi Assano Stangler, Heloísa Ferro, Julia Calisto, Josiane Brehm, Gabriel Felicio Morais, Camila Hartmann, Murilo Guedes

**Affiliations:** 1 Internal Medicine Department, Federal University of Paraná, Curitiba, BRA; 2 Medicine, Pontifícia Universidade Católica do Paraná, Curitiba, BRA; 3 Internal Medicine/Cardiology, School of Medicine, Pontifícia Universidade Católica do Paraná, Curitiba, BRA; 4 Internal Medicine/Cardiology, Marcelino Champagnat Hospital, Curitiba, BRA; 5 Internal Medicine, School of Medicine, Pontifícia Universidade Católica do Paraná, Curitiba, BRA; 6 Cardiology, School of Medicine, Pontifícia Universidade Católica do Paraná, Curitiba, BRA; 7 Cardiology, Marcelino Champagnat Hospital, Curitiba, BRA; 8 Internal Medicine, Pontifícia Universidade Católica do Paraná, Curitiba, BRA

**Keywords:** in-hospital, mortality, risk factor, acute coronary syndrome, kidney disease

## Abstract

Background

An acute coronary syndrome (ACS) event can be linked to several risk factors, including kidney disease. Currently, it is unknown if kidney disease is associated with the in-hospital mortality of patients admitted with ACS, regardless of the main confounders. In this study, we aimed to determine if kidney disease predicts in-hospital mortality among ACS patients.

Methodology

This is a retrospective cohort study that included patients who were admitted to the cardiology center with ACS. The patients were analyzed for their clinical characteristics, previous diseases, risk factors, and blood samples for laboratory analysis. Continuous variables were analyzed using Student’s t-test, and categorical variables using the chi-square test. A p-value of <0.05 was considered statistically significant.

Results

Of the 340 patients who were included in the study, 59 had ST-elevation myocardial infarction. The mean age of the patients was 62.17 years, 59.41% were male, 67.9% were Caucasian, 26% had diabetes, and 20% had a history of coronary artery disease. Age, systolic blood pressure, and a history of myocardial infarction and chronic kidney disease were linked with a higher mortality rate. In the multivariate analysis, only kidney disease was shown to be an independent marker of mortality.

Conclusions

Among individuals admitted with ACS, kidney disease at hospital admission is associated with increased chances of in-hospital mortality, regardless of other major and minor cardiovascular comorbidities and inflammation at baseline.

## Introduction

Cardiovascular diseases are the main cause of mortality worldwide. While their incidence has been increasing in developing countries, it has been decreasing in developed countries in the last decades [1]. Almost half of the deaths are caused by coronary heart disease that follows acute coronary syndrome (ACS). It is most prevalent in the emergency room as ACS events, which presents as the rupture or erosion of the atherosclerotic plaque followed by thrombosis and local inflammation [2].

Despite the rise in the incidence of cardiovascular diseases, the lethality of ACS has been decreasing due to several factors such as the intensification of statin therapy, the more frequent use of antiplatelet medication, improvement in health services and modern interventional therapies, and better intensive care and supportive therapies [3,4]. However, studies continue to search for new insights into the factors that influence this higher patient population [5].

The factors that are linked to higher cardiovascular risk and higher mortality rate in acute events are also related to systemic inflammatory changes, oxidative stress, vascular calcification, and prothrombotic states [2].

Kidney disease causes a systemic and chronic proinflammatory state that contributes to the vascular and cardiac remodeling process, resulting in atherosclerotic lesions, vascular calcification, and senescence, as well as myocardial fibrosis and calcification of the heart valves. Thus, even though factors such as diabetes, systolic hypertension, smoking, little to no physical activities, alcohol abuse, and left ventricular hypertrophy are cardiovascular mortality predictors in patients with kidney disease, chronic kidney disease (CKD) is by itself an important marker of cardiovascular risk [2].

Several related mechanisms have been described for the association between CKD and cardiovascular risk, including oxidative stress, inflammation, vascular calcification, and iron metabolism [6,7]. However, few studies show kidney disease as an indicator of mortality in patients presenting with ACS. Recently, a study found a correlation between CKD and a worse long-term prognosis in ACS [8], although when adjusted for other confounders, the association was not clinically important.

Therefore, we propose that kidney disease can not only be a cardiovascular risk factor for outpatients with coronary artery disease but also a marker of cardiovascular in-hospital mortality among individuals who develop ACS. With this study, we aim to evaluate if kidney disease diagnosed at hospital admission is associated with a higher mortality rate in patients with ACS who are admitted to the cardiology department, regardless of potential confounders.

## Materials and methods

This is a retrospective cohort study that evaluates patients admitted to a Brazilian hospital with a confirmed diagnosis of ACS, with or without ST-segment elevation, from 2012 to 2019. The institutional review committee on human research approved the study (protocol number 31349420.0.0000.0020).

The inclusion criteria were patients with a confirmed diagnosis of ACS and available records of admission examinations in the first 12 hours, including blood count, troponin, creatinine, C-reactive protein, sodium, and potassium, as well as clinical data such as comorbidities and risk factors.

Diagnosis of ACS was defined by the physician at admission. Glomerular filtration rate (GFR) was estimated from creatinine values using the CKD-EPI equation, and kidney disease was defined by GFR of <60 mL/minute/1.73 m^2^. Patients with incomplete medical records or those diagnosed with ACS during hospitalization were excluded.

Upon admission, patients’ gender, age, heart rate, and blood pressure data were collected. The risk factors captured by medical intervention at the time of admission included hypertension, dyslipidemia, diabetes, smoking, obesity and alcoholism, history of a previous cardiovascular event, presence of known coronary artery disease, and peripheral vascular disease. All comorbidities were captured using the electronic health record of the participants. Additionally, blood samples were also collected for laboratory analysis.

Statistical analysis

Data were analyzed using SPSS, version 25 (IBM Corp., Armonk, NY, USA). Descriptive characteristics were calculated for continuous variables (median and interquartile range, IQR) and categorical variables (frequency/percentage). Continuous variables were analyzed using Student’s t-test, whereas categorical variables were analyzed using the chi-square test. To evaluate the association between kidney disease and in-hospital mortality, a multivariate model was developed. Covariates for the multivariate analysis were selected based both on clinical relevance according to a priori assumptions and if they remained statistically significant in the univariate analysis. A p-value of <0.05 was considered statistically significant.

## Results

Between 2012 and 2019, a total of 756 patients were admitted to the hospital with chest pain or suspected ACS. Of these patients, 449 were excluded for not meeting the inclusion criteria. Finally, 340 patients were included in this study, of which 202 were males (59.4%) and 138 were females (40.6%), with an average age of 62.2 years.

Table 1 shows the heart rate, blood pressure, and laboratory findings upon admission. Table 1 also shows the population characteristics so that we can outline our study sample and correlate it to the mortality outcome. Table 2 describes patients’ characteristics stratified by in-hospital mortality. Among patients who did not survive during hospitalization, the mean age was 80.43 (±3.22) years, the average systolic blood pressure was 112.71 (± 17.76) mmHg, and there were four cases of myocardial infarctions, five cases of stable angina, and three cases of kidney disease. For those who survived, the mean age was 61.77 (± 0.9) years, the average systolic blood pressure was 135.42 (± 1.59) mmHg, and there were 62 cases of myocardial infarction, 88 cases of stable angina, and 18 cases of kidney disease.

**Table 1:** Variables at the time of admission. *Average (standard deviation); #frequency (percentage) STEMI: ST-elevation myocardial infarction; AMI: acute myocardial infarction

Variable	
Age (years)	62.2 (16.5)*
Heart rate (beats per minute)	79.2 (22.1)*
Systolic blood pressure (mmHg)	134.9 (8.5)*
Hemoglobin (g/dL)	13.4 (2.2)*
Laboratory tests on admission	17.4 (36.6)*
Serum sodium (mEq/L)	136.7 (10.0)*
Potassium (mmol/L)	4.1 (0.6)*
C-reactive protein (CRP) (mg/dL)	1.4 (4.1)*
Urea (mg/dL)	41.2 (24.7)*
Male	202 (59.4%)#
STEMI	59 (17.4%)#
Hypertension	207 (60.9%)#
Dyslipidemia	205 (60.3%)#
Diabetes	237 (69.7%)#
Previous AMI	258 (75.9%)#
COPD/Pneumopathies	297 (87.4%)#
Obesity	224 (65.9%)#
Alcoholism	286 (84.1%)#
Smoking	270 (79.4%)#

**Table 2:** Variables according to the outcome of mortality. STEMI: ST-elevation myocardial infarction

Variable	Non-survivor	Survivor	P-value
Gender (male)	2	200	0.10
Age (years)	80.43 (±3.22)	61.77 (±0.9)	0.07
Heart rate (beats per minute)	105 (±13.12)	78.59 (±1.22)	0.06
STEMI (yes)	3	4	0.72
Systolic blood pressure (mmHg)	112.71 (±17.76)	135.42 (±1.59)	0.03
Hemoglobin (g/dL)	11.11 (±0.72)	13.48 (±0.14)	0.80
Troponin-I (ng/dL)	6.54 (±4.41)	1.89 (±0.61)	0.26
C-reactive protein (CRP) (mg/dL)	44.58 (±26.78)	16.29 (±2.64)	0.03
Serum sodium (mEq/L)	136.28 (±11.37)	136.68 (±0.7)	0.80
Potassium (mmol/L)	4.78 (±0.59)	4.11 (±0.03)	0.00
Serum creatinine (mg/dL)	3.67 (±1.15)	1.30 (±0.28)	0.36
Urea (mg/dL)	112.6 (±20.06)	38.65 (±1.34)	0.00
Hypertension (yes)	6	201	0.20
Dyslipidemia (yes)	4	109	0.20
Diabetes (yes)	4	82	0.08
Cancer (yes)	0	28	0.52
Previous myocardial infarction (yes)	4	62	0.03
Previous stroke (yes)	0	25	0.55
Kidney disease (yes)	3	18	0.00
Smoking (yes)	1	49	0.70

In the multiple logistic regression analysis, kidney failure, C-reactive protein, previous myocardial infarction, and systolic blood pressure were included, with p-values of 0.028, 0.145, 0.089, and 0.193; odds ratios of 4.82, 2.12, 2.90, and 1.69; and 95% confidence intervals of 1.23-43.51, 0.99-1.02, 0.80-22.67, and 0.95-1.00, respectively. According to the analysis, age, systolic blood pressure, previous myocardial infarction, and history of kidney disease were associated with a higher mortality rate (Table 3). Only kidney disease was an independent marker of mortality (Table 3).

**Table 3:** Multivariate analysis. CRP: C-reactive protein; AMI: acute myocardial infarction; HR: hazard ratio; CI: confidence interval

Variable	P-value	HR	95% CI
Kidney disease	0.028	4.82	1.23–43.51
CRP (mg/dL)	0.145	2.12	0.99–1.02
Previous AMI	0.089	2.90	0.80–22.67
Systolic blood pressure	0.193	1.69	0.95–1.00

## Discussion

In this single-center retrospective cohort study of patients who were admitted to the hospital due to ACS, kidney dysfunction was associated with a four-fold increase in mortality during hospitalization. This association was noted even after adjusting for clinically important confounders such as previous cardiovascular events, hypertension, and inflammation.

The causal role of kidney diseases, particularly CKD, on the development of cardiovascular diseases is well established. Robust observational studies have shown that GFR and albuminuria are strong risk factors for cardiovascular mortality, and interventional studies have confirmed that reducing the progression of CKD reduces major adverse cardiovascular events [9]. The association between heart and kidney outcomes has recently been highlighted by the successful development of a broad group of interventions targeting both systems for outpatient management, for example, FIGARO [10] and FIDELIO [11] trials. These studies have indicated that finerenone impacts cardiovascular outcomes in patients with type 2 diabetes and CKD in a background of maximal renin-angiotensin system blockade therapy, primarily due to a reduction in hospitalization due to heart failure. Furthermore, McMurray et al. and Heerspink et al. showed that among patients with CKD (in the presence or absence of diabetes), the risk of decline in estimated GFR of at least 50%, end-stage kidney disease, or death from renal or cardiovascular causes were significantly lower with dapagliflozin than with placebo.

In the inpatient setting, however, the interplay between kidney diseases (either acute or chronic) and cardiovascular events is not well described. Particularly, among individuals with ACS, the impact of kidney disease has not been addressed, regardless of the main confounders. According to the study by Dohi et al., kidney disease is a powerful determinant of long-term all-cause and cardiovascular mortality after ACS [14]. Several other studies have also identified CKD as a powerful independent predictor of adverse outcomes after percutaneous coronary intervention and coronary artery bypass grafting [15,16].

Several factors can explain our findings such as the overlap of risk factors between kidney disease and coronary artery disease, worse inflammation and oxidative stress in kidney disease patients, as well as accelerated vascular calcification [17]. Other factors to consider include a higher incidence of secondary bleeding due to antiplatelet medication, as well as the susceptibility to injuries induced by contrast, usually used in coronary angiograms [18-20].

This study has several limitations. First, because this is a retrospective cohort, confounding cannot be ruled out in our model. Second, the external validity of our findings may be limited because this is a single-center study, which can introduce selection bias in the sample; therefore, the results may not be generalizable to other populations. Finally, information bias is a potential limitation, given that serum creatinine was measured at hospital admission, and the exposure could not be clearly defined as chronic or acute kidney disease.

Despite these limitations, this observational study has several strengths. Enrolled individuals were investigated for important confounders of kidney disease, such as previous cardiovascular events, and inflammatory surrogates, such as C-reactive protein levels. Additionally, ACS was defined by the clinical diagnosis according to clear and validated criteria.

## Conclusions

Among individuals admitted with ACS, kidney disease at hospital admission is associated with increased chances of in-hospital mortality, regardless of cardiovascular comorbidities and inflammation at baseline. These findings may help the development of prognostic scores for ACS risk stratification, as well as to guide further interventions targeting risk reduction for kidney disease in this population.
